# Assessing Attitude Toward COVID-19 Vaccination in South Korea

**DOI:** 10.3389/fpsyg.2021.694151

**Published:** 2021-07-28

**Authors:** Shiva Raj Acharya, Deog Hwan Moon, Yong Chul Shin

**Affiliations:** ^1^Graduate School of Public Health, Inje University, Busan, South Korea; ^2^Department of Occupational Health and Safety, Inje University, Gimhae, South Korea

**Keywords:** COVID-19 vaccine, attitudes, perceptions, vaccine acceptance, safety

## Abstract

Vaccines are the most effective strategy to safeguard against COVID−19 and it is crucial to assess community acceptance of COVID-19 vaccination. This exploratory study aimed to assess the attitude of immigrants toward the acceptance of COVID-19 vaccines in South Korea. A web-based anonymous study was completed by 463 immigrants. The data were statistically analyzed using a logistic regression model and ANOVA test. On a scale of 0–6, the average attitude toward the COVID-19 vaccination was 4.17 ± 1.73, indicating generally positive attitudes. The proportion of the immigrants who were certain to get COVID-19 vaccination was 55.3%. Only 36.7% reported that the COVID-19 vaccines are safe. Of the immigrants, 72.6% showed high acceptance and 27.4% low acceptance toward the COVID-19 vaccines. Vaccine safety concern was the major predictor for COVID-19 vaccine acceptance. Up-to-date, valid information on COVID-19 vaccine safety, and vaccine risk communication strategies are required to increase vaccine acceptability.

## Introduction

Vaccines are the most effective strategy to protect the population from COVID−19. It is critical to assess community acceptance of COVID-19 vaccination now since COVID-19 vaccination has started globally (Islam et al., 2021). Although data suggest that the approved vaccines are safe and effective, long-term effectiveness and side effects are unknown. Understandably, the general public are uncertain of the current vaccine’s acceptance (Mannan and Farhana, 2020; Machida et al., 2021). Immigrant groups are not homogeneous, and their experiences of and attitudes toward vaccines vary greatly. Globally, immigrants are at increased risk of COVID-19 infection due to factors such as ongoing stigma and discrimination, economic disenfranchisement, and barriers to public health care. Limited studies have looked at individual vaccine uptake results by minorities including associated factors with any differences in vaccine acceptance (Caserotti et al., 1982; Mannan and Farhana, 2020; Islam et al., 2021; Thomas et al., 2021).

Although the most efficient way of controlling the spread of the virus is to protect oneself from being exposed to COVID-19, it is very important to vaccinate the community’s vulnerable group, such as immigrants (Acharya et al., 2020; Mannan and Farhana, 2020; Lake et al., 2021). A study found that only 54% of the respondents said that they intended to have the vaccination (Lin et al., 2020). Furthermore, a global survey of potential COVID-19 vaccine acceptance showed that 48% of their study population were uncertain and unsure about the acceptance of COVID-19 vaccination (Lazarus et al., 2021). These relatively low proportions of people willing to get vaccinated is a serious issue (Schaffer DeRoo et al., 2020).

Vaccination against COVID-19 is voluntary in Korea therefore it’s critical to assess the existing attitudes of specific communities in order to have a successful vaccine campaign. At the time of writing (February 2021) in South Korea, both the AstraZeneca and Pfizer vaccines have received approval and are currently in use in the country’s vaccination programme (Korea Disease Control and Prevention Agency, 2021). People’s attitudes and perceptions about COVID-19 are critical for government and policymakers in addressing many of the hurdles to vaccination in such a setting. Vaccine hesitancy may pose a significant barrier in the COVID-19 immunization campaign (Al-Qerem and Jarab, 2021; Bhartiya et al., 2021). However, herd immunity requires a certain percentage of the population to be vaccinated. This goal is unlikely to be achieved unless the immigrant community also gets fully vaccinated (COCONEL Group, 2020; Schaffer DeRoo et al., 2020). In Korea, the perspective on the COVID-19 vaccines has not been studied, and it is expected that vaccine-related attitudes will be very diverse based on demographic factors, ethnic group, knowledge regarding COVID-19 and the vaccine’s availability. COVID-19 vaccine risk communication during the vaccine distribution timeline, and prioritization of the group for vaccination programs, have been identified as a major concern around the world (Kerr et al., 2021; Warren and Lofstedt, 2021). To prevent differences in vaccination reluctance, different communication tools are necessary within and between communities (Larson et al., 2015).

Furthermore, immigrants encounter hurdles to adequate access to health care services due to risk communication in culture, language, and economic conditions which inadvertently influenced their attitudes toward vaccination. In the context of vaccine risk communication, COVID-19 risk communication has varied widely (Warren and Lofstedt, 2021). Since globally immigrants were blamed for the coronavirus transmission, risk communication plays an important role in any risk management plan, particularly in light of the COVID-19 pandemic (Kerr et al., 2021; Thomas et al., 2021). Immigrants are unlikely to be well informed and tend not to access to health care enough leading to specific COVID-19 vaccination attitudes (Galanis et al., 2013). To date, there is no prior study on attitudes toward the COVID-19 vaccine among immigrants in South Korea. In this study, we analyze the attitude of immigrants toward the COVID-19 vaccination.

## Materials and Methods

### Setting and Sampling

This was a cross-sectional study conducted among 463 immigrants in South Korea through an anonymous internet-based survey. The study was conducted between 25 January and 10 February 2021. A semi-structured questionnaire was designed with a google survey tool and disseminated publicly on the various social platforms (Facebook pages/groups for immigrants, LinkedIn) and was also shared personally (WhatsApp, Viber, Email, KakaoTalk) with the study population. The survey questionnaire was conducted in the English language. The majority of the participants (421) responded through social platforms. Immigrants over 18 years old, living in Korea for more than 1 year and who were able to provide consent were included in the study. The sample size was calculated by using the formula: *n* = Z^2^pq/d^2^ (Hasan et al., 2021).

*n- desired sample size for the study*

*Z- the standard normal variate which corresponds to 95% confidence interval*

*p- proportion of the estimated population = 50%*

*q- 1-p = 0.5*

*d- precision = 0.05*.

As there is no study available on attitude toward the COVID-19 vaccines in South Korea focusing on immigrants, with a proportion (p) of 50%, at a confidence interval of 95% and a 10% non-response rate, a sample size of 424 was estimated. Our sample size exceeded this estimate. In total, 468 participants participated in the study, but only 463 participants provided the consent for the study.

### Measures

#### Sociodemographic Variables

Participants reported their sex, age, vaccination history, marital status (single/married), region (Asia/Europe/Africa/North & South America), residential area (capital/non-capital), and living type (alone/with family), education status (college/university level) and income (<3000$/ > 3000$).

#### Attitude Assessment Toward COVID-19 Vaccination

The attitude section consisted of validated three-items (Hogan et al., 2020; Bhartiya et al., 2021; Islam et al., 2021; Seale et al., 2021; Verger et al., 2021) (e.g., *The COVID-19 vaccines are safe; I will get the COVID-19 vaccine without any hesitation*) and the response of each item was indicated on a three-point scale (*0 = Disagree, 1 = Probably*, and *2 = Agree*). The scores obtained from all three questions per respondent were added to obtain the attitude score (Cronbach’s alpha: 0.76, range: 0–6). For the purpose of logistic regression, we further categorized the attitude scores based on the median to interpret the “COVID-19 vaccine acceptance” as high acceptance (≥4) and low acceptance (<4).

### Data Collection and Statistical Analysis

The data were collected online due to the strict social distancing measures in effect at that time. The data were analyzed using IBM SPSS 23.0. Descriptive statistical analysis (means, standard deviations) was performed for basic information of immigrants and attitude responses. ANOVA test was performed to determine significant relations of the mean attitudes scores with socio-demographic information. Finally, logistic regression was used to investigate the factors associated with COVID-19 vaccine acceptance among immigrants categorized into low and high acceptance levels based on attitude scores. All statistical tests were considered significant at a 95% confidence interval with a *P*-value < 0.05. Ethical consideration was taken from the ethical review board of Inje University. Participants received electronic informed consent to complete the online questionnaire which appeared on the first page of the survey.

## Results

Among the 463 immigrants, most were above 25 years (81.6%), male (67.8%) and married (61.3%). Majorities of the immigrant (74.7%) were from Asian countries. More than half of the immigrants were living alone (55.9%) and had college or below levels of education (58.7%). The vast majority of participants (90.5%) reported they received all other recommended vaccines in their lifetime (e.g., measles vaccine) (Table 1).

**TABLE 1:** General characteristic of participants and distribution of each attitude items.

Variables	Frequency (%) (*n* = 463)		
**Age (years)**			
18–25	85 (18.4)		
>25	378 (81.6)		
**Gender**			
Male	314 (67.8)		
Female	149 (32.2)		
**Education**			
College or below	272 (58.7)		
University or above	191 (41.3)		
**Family type**			
Alone	259 (55.9)		
Family	204 (44.1)		
**Residence**			
Capital	221 (47.7)		
Non-capital	242 (52.3)		
**Income**			
<3000$	346 (81)		
>3000$	88 (19)		
**Marital status**			
Single	179 (38.7)		
Married	284 (61.3)		
**Region**			
Asia	346 (74.7)		
Europe & Australia	37 (8)		
North America	14 (3)		
South America	38 (8.2)		
Africa	28 (6)		
**Vaccination history (received all necessary vaccines in your life?)**			
Yes	419 (90.5)		
No	44 (9.5)		
**Vaccine developed during an epidemic/pandemic could not be considered guaranteed**			
Agree	170 (36.7)		
Disagree	293 (63.3)		
**Attitude questions**	** Agree**	** Probably**	** Disagree**
COVID-19 Vaccines are safe	170 (36.7)	257 (55.5)	36 (7.8)
I will take the COVID-19 vaccine without any hesitation	256 (55.3)	123 (26.6)	84 (18.1)
I will recommend to my family/friends/relatives to get vaccinated	300 (64.8)	99 (21.4)	64 (13.8)

The mean score of attitudes toward the COVID-19 vaccines was 4.17 ± 1.73 on a scale of 0–6. About a third (36.7%) of participants indicated that a vaccine developed during an epidemic/pandemic situation could not be considered guaranteed and reported that the COVID-19 vaccines are safe. Meanwhile, 7.8% disagreed with the statement that the COVID-19 vaccines are safe. More than half of the immigrants (55.3%) agree to be vaccinated with the COVID-19 vaccine without any hesitation whereas 64.8% of immigrants mentioned that they will surely recommend the COVID-19 vaccination to their family and friends (Table 1). The mean score of attitudes was significantly higher among immigrants who reported being not married (Table 2). Of the sampled population, 72.6% (*n* = 336) showed high acceptance and 27.4% (*n* = 127) low acceptance toward the COVID-19 vaccines. The Immigrants who agreed to the statement “vaccine developed during an epidemic/pandemic situation could not be considered guaranteed” were 1.7 times (95% CI: 1.084–2.673) more likely to get COVID-19 vaccination (Table 3).

**TABLE 2:** Group difference analysis with attitudes toward COVID-19 vaccination.

Variables	Attitudes toward the COVID-19 vaccine		
	*Mean (S.D)*	*t/F*	*P-value*
**Age (years)**			
18–25	4.18 (1.79)	0.01	0.973
>25	4.17 (1.71)		
**Gender**			
Male	4.25 (1.67)	1.97	0.161
Female	4.01 (1.82)		
**Education**			
College or below	4.07 (1.79)	2.24	0.135
University or above	4.31 (1.63)		
**Family type**			
Alone	4.15 (1.77)	0.51	0.821
Family	4.19 (1.68)		
**Residence**			
Capital	4.17 (1.64)	0.01	0.988
Non-capital	4.17 (1.80)		
**Income**			
<3000$	4.15 (1.74)	0.29	0.585
>3000$	4.26 (1.68)		
**Marital status**			
Single	4.37 (1.57)	4.06	0.044*
Married	4.04 (1.81)		
**Region**			
Asia	4.20 (1.69)	1.12	0.344
Europe & Australia	4.22 (1.91)		
North America	3.21 (2.25)		
South America	4.26 (1.78)		
Africa	4.14 (1.53)		
**Vaccination history (received all necessary vaccines in your life?)**			
Yes	4.17 (1.75)	0.02	0.964
No	4.18 (1.46)		

*Statistically significant at *p* < 0.05.

**TABLE 3:** Logistic regression analysis with low and high acceptance toward COVID-19 vaccine.

Factors	COVID-19 vaccine acceptance^a^				
	*Low*	*High*	*B*	*P-value*	*OR (95% CI)*
**Gender (ref: female)**					
Male	85 (27.1)	229 (72.9)	0.044	0.847	1.045(0.667–1.638)
Female	42 (28.2)	107 (71.8)			
**Age (ref: > 25 years)**					
18–25	21 (24.7)	64 (75.3)	0.096	0.747	1.100(0.616–1.966)
>25	106 (28)	272 (72)			
**Residence (ref: non-capital)**					
Capital	61 (27.6)	160 (72.4)	−0.055	0.800	0.947(0.621–1.444)
Non-capital	66 (27.3)	176 (72.7)			
**Marital status (ref: married)**					
Single	40 (22.3)	139 (77.7)	0.377	0.120	1.457(0.907–2.342)
Married	87 (30.6)	197 (69.4)			
**Living type (ref: with family)**					
Alone	74 (28.6)	185 (71.4)	−0.254	0.245	0.776(0.506–1.190)
With family	53 (26)	151 (74)			
**Education (ref: university or above)**					
College or below	82 (30.1)	190 (69.9)	−0.289	0.211	0.749(0.476–1.178)
University or above	45 (23.6)	146 (76.4)			
**Income (ref: 3000$)**					
<3000$	105 (28)	270 (72)	−0.059	0.838	0.943(0.538–1.652)
>3000$	22 (25)	66 (75)			
**Vaccination history (received all necessary vaccines in your life?) (ref: no)**					
Yes	115 (27.4)	304 (72.6)	−0.039	0.914	0.961(0.472–1.961)
No	12 (27.3)	32 (72.7)			
**Vaccine developed during an epidemic/pandemic could not be considered guaranteed (ref: disagree)**					
Agree	36 (21.2)	134 (78.8)	0.532	0.021*	1.702(1.084–2.673)
Disagree	91 (31.1)	202 (68.9)			

*Statistically significant at *P* < 0.05. ^*a*^High acceptance indicates surely get vaccinated and will surely recommend vaccinations to family and friends; low acceptance indicates maybe self-vaccinate and probably/maybe recommend the vaccine to family and friends.

## Discussion

Understanding the epidemiological aspects of disease control, as well as the efficacy and progress of the vaccination program, demands an understanding of the local population’s attitudes and practices toward the COVID-19 vaccines. Our study aims to highlight the attitude regarding the COVID-19 vaccines, and also the predictors of vaccine acceptance among immigrants in South Korea.

Our study shows more than half of the study participants (55.3%) were willing to take the COVID-19 vaccines. In comparison, a survey from China reveals only about 28.7% reported a definite intention (Lin et al., 2020). A study showed a higher COVID-19 vaccine intention in Malaysia (94.3%) (Wong et al., 2020) of which 48.2% reported a definite intention, Indonesia (67%) (Harapan et al., 2020) and Japan (67.1%) (Machida et al., 2021). Also, an online survey also found a higher vaccine intention in France (74%) (COCONEL Group, 2020), United States (74.1%) (Hogan et al., 2020) and Europe (73%) (Neumann-Böhme et al., 2020).

According to a global survey of 19 nations, 71.5% of the participants said they would get the COVID-19 vaccine if available (Lazarus et al., 2021) which is higher than the results of our analysis. This may be attributed to cultural differences between South Korea and the rest of the world (Lee et al., 2021). Vaccine acceptance may need to be improved further, as high vaccination coverage is needed to combat epidemics (Machida et al., 2021). Vaccine reluctance among not only the general public but also medical practitioners has become a concern in recent years. Vaccine reluctance varies by time, location, and vaccine type, and is affected by a range of factors (Caserotti et al., 1982; Machida et al., 2021; Verger et al., 2021). Therefore, to arrange promotional activities to improve vaccine acceptance, it is vital to ascertain vaccine acceptance of the COVID-19 vaccines and the factors that influence it in each location.

A range of reports has shown that up to 40% of the general population had unfavorable views about potential COVID-19 vaccines (Bhartiya et al., 2021; Lake et al., 2021; Verger et al., 2021). One of the principal factors behind these attitudes seems to be a concern that the new vaccines will not be safe (Lin et al., 2020; Verger et al., 2021). In this study period almost three-quarters of the respondents (72.6%) reported high acceptance of the COVID-19 vaccine which is considerably higher than the European study (48.6%) (Verger et al., 2021). In our work, the perception that vaccines developed in an emergency cannot be guaranteed safe appeared to be significantly associated with the acceptance of COVID-19 vaccines (Verger et al., 2021). The key factor found to be consistent with COVID-19 vaccination acceptance was the vaccine’s safety and effectiveness, which has also been documented in other research related to the new vaccine. The observed result indicates that these outcomes are reliable and, most importantly, that they encompass contextual cultural, educational, and social factors (Caserotti et al., 1982; Islam et al., 2021; Machida et al., 2021; Verger et al., 2021).

Even in well-established vaccination systems, vaccine reluctance remains a major obstacle to population vaccination. There may also be specific vaccine factors or misinformation that make a vaccine more or less acceptable to certain groups (Lazarus et al., 2021). Before and during vaccine rollout, practical ways to eliminate vaccination barriers in immigrant populations must be implemented, including effective communication and supervision. Cultural considerations, differing understanding and attitudes about disease causes, and healthcare access issues are all hurdles to vaccination faced by immigrant populations (Thomas et al., 2021). In the following periods, it is essential to regularly monitor the attitudes and practices of all specific groups of the community toward the COVID-19 vaccines. COVID-19 vaccines should be prioritized for disadvantaged populations due to the strong demand (COCONEL Group, 2020; Lazarus et al., 2021).

## Limitations

There are certain drawbacks to this research. Since it was a cross-sectional study, so the causality cannot be attributed to the findings in the regression models. This was a web-based analysis with the potential for bias. The findings of our study demonstrate immigrants’ attitudes toward the COVID-19 vaccine. This result might be different in the general population.

## Conclusion

Interventional programs targeting vulnerable populations with a higher risk of vaccine reluctance are most crucial to minimize the poor vaccination rates. Nevertheless, this is the first-ever study of immigrant’s perspectives about the COVID-19 vaccination in South Korea which will be very crucial for health policymakers and government to address proper vaccination among the vulnerable and neglected group of communities to mitigate the impacts of the pandemic. Adequate knowledge, positive attitudes and perceptions toward COVID-19 vaccination should be ensured to reduce vaccine hesitancy. Regular monitoring of the vaccination program and vaccination risk communication strategies are essential to ensure trust in COVID-19 vaccines. Furthermore, to minimize vaccine reluctance, a variety of vaccination methods targeting COVID-19 vaccine risk communication should be implemented adequately.

## Data Availability Statement

The original contributions presented in the study are included in the article/supplementary material, further inquiries can be directed to the corresponding author.

## Ethics Statement

The studies involving human participants were reviewed and approved by the Institutional Review Board of Inje University. The patients/participants provided their written informed consent to participate in this study.

## Author Contributions

SA interpreted the study data, analysis, and manuscript writing. YS and DM performed research concept and literature discussion. All authors contributed to the article and approved the submitted version.

## Conflict of Interest

The authors declare that the research was conducted in the absence of any commercial or financial relationships that could be construed as a potential conflict of interest.

## Publisher’s Note

All claims expressed in this article are solely those of the authors and do not necessarily represent those of their affiliated organizations, or those of the publisher, the editors and the reviewers. Any product that may be evaluated in this article, or claim that may be made by its manufacturer, is not guaranteed or endorsed by the publisher.
